# Editorial: Advances on innovative protein-based complexes with promising functionality, nutrient retention and encapsulation capacity

**DOI:** 10.3389/fnut.2023.1217964

**Published:** 2023-05-30

**Authors:** Shuai Chen, Xiao Feng, Xi Yu

**Affiliations:** ^1^School of Public Health, Wuhan University, Wuhan, China; ^2^School of Food Science and Engineering, Nanjing University of Finance and Economics, Nanjing, China; ^3^Faculty of Medicine, Macau University of Science and Technology, Macau, Macau SAR, China

**Keywords:** protein-based complex, encapsulation, emulsion, protein functionality, nutrient retention

Protein-based complexes are of great interest in food industry and research, such as protein gels, protein-stabilized emulsions, protein-based carriers of bioactive compounds, etc. Proteins derived from different resources with different processing technology have different structure and properties, which enables their application in nutrient retention, delivery, improved digestibility and other functionalities. Emerging technologies, micro- and macronutrients, and new protein resources have been exploited for protein-based complex research. This Research Topic aims to provide a platform for researchers to share their state-of-the-art innovations on the preparation, evaluation and application of protein-based complexes, especially those with improved encapsulation efficiency, nutrient retention, anti-nutritional factor removal, etc., compared to currently available work. In addition to emerging technologies, innovative proteins (e.g., insect, yeast, pseudocereal), hydrocolloids, soluble fibers, and ligand receptors are also of great interest. This Research Topic includes the development of innovative protein-based complexes, improved functionality of protein-based complexes, protein-based complexes for nutrient delivery, nutrient preservation of protein-based complexes during food processing and *in vitro* digestion, reduction of antinutrients and unpleasant compounds from protein-based foods.

The binding behavior of protein and bioactive compound can affect the functional properties of their complexes. For example, β-carotene improved DPPH radical scavenging activity, foaming capacity, and emulsifying stability of soybean protein isolate (Zhang, Zhao et al.). The binding with quercetin changed the secondary structure of soybean protein isolate, resulting in a partially unfolded and more flexible structure. Meanwhile, modification by quercetin enhanced the foaming and emulsifying capacities of soybean protein isolate (Zhang, Hou et al.). The addition of polysaccharides had an important effect on the properties and functions of protein-based complexes. Chitosan oligosaccharide modification not only delayed the oxidation of myofibrillar protein but also promoted myofibrillar protein to show better solubility, foaming, and foaming stability (Cong et al.). Dong et al. found that the addition of octenyl succinic anhydride modified starch (OSAS) improved hydrophobicity and emulsifying capacity of the soy protein (SP)-(-)-epigallocatechin-3-gallate (EGCG) complexes. Emulsion gels is a novel complex that prepared from proteins, polysaccharides and lipids, which can be used as solid fat replacers. κ-carrageenan (κC), high-acyl gellan (HA), konjac glucomannanon (KGM), pea protein isolate (PPI) and sunflower seed oil were used to prepare the emulsion gels (Hou et al.). The presence of polysaccharides enhanced the hardness, storage modulus and resistance against deformation of emulsion gel, where PPI/κC system exhibited superior hardness with a similar level of pig back fat.

Some authors reported novel application scenarios for various proteins. Yan et al. reviewed the application of zein-based nano-systems for the delivery of bioactive compounds. Recent advances on using zein-based encapsulation and protection systems were systematically reviewed and discussed, offering insights and inspirations for future developers. Wang et al. made improvements on whey protein with the aid of hesperidin, to enhance their performances as emulsion stabilizer. The antioxidant capacity of the complex was improved as well. The work offers new thoughts on the combined and synergistic utilization of proteins and natural bioactive compounds.

Some of the papers investigated the undiscovered functions of traditional proteins. Sun, Xu et al. revealed that fermented soymilk has antioxidative and anti-inflammatory effect on mice, providing references on the utilization of fermented products of traditional proteins. The in-depth characterization is also important for the investigation of these large biomolecules. The structure-function relationship can be better understood with the help of modern imaging techniques. Sun, Li et al. reviewed the recent advances on the characterization of various biomacromolecules in foods with the aid of small-angle X-ray scattering, summarizing the pros and cons of this advanced imaging technique for unveiling the mystery of various large molecules in food materials.

## Author contributions

SC, XF, and XY prepared, checked and revised the manuscript, and approved the submitted version. All authors contributed to the article and approved the submitted version.

